# Cost-effectiveness of tipranavir versus comparator protease inhibitor regimens in HIV infected patients previously exposed to antiretroviral therapy in the Netherlands

**DOI:** 10.1186/1478-7547-5-15

**Published:** 2007-11-22

**Authors:** Gijs AA Hubben, Jasper M Bos, Christa A Veltman-Starkenburg, Simon Stegmeijer, Henrik W Finnern, Bregt S Kappelhoff, Kit N Simpson, Andrea Tramarin, Maarten J Postma

**Affiliations:** 1 University Center for Pharmacy, University of Groningen, The Netherlands; 2 University Medical Center Groningen (UMCG), The Netherlands; 3 Boehringer Ingelheim GmbH, Ingelheim, Germany; 4 Boehringer Ingelheim BV, Alkmaar, The Netherlands; 5 Pharmacy and Clinical Sciences, Medical University of South Carolina, Charleston, South Carolina, USA; 6 Agenzia Regionale Socio Sanitaria del Veneto, Venice, Italy

## Abstract

**Background:**

This study compares the costs and effects of a regimen with ritonavir-boosted tipranavir (TPV/r) to a physician-selected genotypically-defined standard-of-care comparator protease inhibitor regimen boosted with ritonavir (CPI/r) in HIV infected patients that were previously exposed to antiretroviral therapy in the Netherlands.

**Methods:**

We compared the projected lifetime costs and effects of two theoretical groups of 1000 patients, one receiving a standard of care regimen with TPV/r as a component and the other receiving a standard of care regimen with CPI/r. A 3-stage Markov model was formulated to represent three different consecutive HAART regimens. The model uses 12 health states based on viral load and CD4+ count to simulate disease progression. The transition probabilities for the Markov model were derived from a United States cohort of treatment experienced HIV patients. Furthermore, the study design was based on 48-week data from the RESIST-2 clinical trial and local Dutch costing data. Cost and health effects were discounted at 4% and 1.5% respectively according to the Dutch guideline. The analysis was conducted from the Dutch healthcare perspective using 2006 unit cost prices.

**Results:**

Our model projects an accumulated discounted cost to the Dutch healthcare system per patient receiving the TPV/r regimen of €167,200 compared to €145,400 for the CPI/r regimen. This results in an incremental cost of €21,800 per patient. The accumulated discounted effect is 7.43 life years or 6.31 quality adjusted life years (QALYs) per patient receiving TPV/r, compared to 6.91 life years or 5.80 QALYs per patient receiving CPI/r. This translates into an incremental effect of TPV/r over CPI/r of 0.52 life years gained (LYG) or 0.51 QALYs gained. The corresponding incremental cost effectiveness ratios (iCERs) are €41,600 per LYG and €42,500 per QALY.

**Conclusion:**

We estimated the iCER for TPV/r compared to CPI/r at approximately €40,000 in treatment experienced HIV-1 infected patients in the Netherlands. This ratio may well be in range of what is acceptable and warrants reimbursement for new drug treatments in the Netherlands, in particular in therapeutic areas as end-stage oncology and HIV and other last-resort health-care interventions.

## Background

The human immunodeficiency virus (HIV) that causes acquired immunodeficiency syndrome (AIDS) confronts us with a pandemic that is one of the biggest health problems in the world today. The Netherlands is known as a low-prevalence country, where the HIV epidemic is mostly confined within risk-groups. In recent years however, the majority of new HIV infections have occurred in patients of the general population without obvious risk-factors. Heisterkamp et al. [1] estimated the number of predominantly HIV-1 infected individuals (regardless of stage of disease) at 8,377 by the end of 2004, in the Netherlands.

The introduction of highly active antiretroviral therapy (HAART) has been one of the greatest therapeutic advances in slowing disease progression of HIV since the introduction of zidovudine in 1987. HAART consists of a combination of nucleoside or nucleotide reverse transcriptase inhibitors (NRTIs), non-nucleoside reverse transcriptase inhibitors (NNRTIs), protease inhibitors (PIs) and/or a fusion inhibitor, acting at different stages of the replication cycle of the virus [2]. These combinations of therapeutics succeed in long-term suppression of viral replication and have led to reduced mortality, improved quality of life and a reduction in hospitalization rates and opportunistic infections [3,4]. However, treatment failure is still a relatively common problem in patients with HIV-1 infection using combination antiretroviral therapy [5]. Contributing factors to treatment failure include poor tolerability, low adherence due to demanding drug regimens and emergence of viral resistance [6,7]. Viral resistance limits the number of therapeutic options available and this effect is amplified when the virus develops cross-resistance. Several studies from the international literature indicate a frequent transmission of drug-resistant strains [8]. In the Netherlands drug-resistant mutations are reported in 10–30% of primary HIV-1 infections [9]. The non-peptidic PI tipranavir (Aptivus^®^, Boehringer Ingelheim) co-administrated with a low dose of ritonavir (TPV/r) provides an additional treatment option for highly treatment-experienced HIV-1 infected patients where the virus developed resistance during the course of previous treatments. TPV/r suppresses viral replication of HIV-1, in particular in isolates that are highly resistant to multiple PIs [10,11].

Two randomized open-label phase III clinical trials, RESIST-1 (Randomized Evaluation of Strategic Intervention in multidrug reSistant patients with Tipranavir) and RESIST-2, have demonstrated that patients treated with TPV/r were twice as likely to achieve a viral load of less than 50 copies per mL compared to investigator-selected genotypically-defined ritonavir-boosted standard-of-care comparator protease inhibitor (CPI/r) therapy at week 48 [12-14]. TPV/r was approved by EMEA and FDA in 2005 for use in highly treatment-experienced HIV-1 infected patients [15].

Economic considerations are of increasing importance for the reimbursement of new therapeutics. In a number of countries (for instance, the Netherlands, United Kingdom, Australia and others), economics evaluations of pharmaceuticals are required for decisions on the reimbursement of such new pharmaceuticals. Several cost-effectiveness studies have been conducted comparing different antiretroviral combinations for HIV. However, in a recent review over 1994–2004 Harling et al. reported that the majority of these studies compare general regimen types (mono, duo and triple HAART therapy) instead of specific therapeutics or combinations of brands [2]. Only a small number of studies compare one antiretroviral to another within the context of HAART. One reason why these evaluations are not frequent might be the difficult task of modeling the complexity of multiple sequential HAART regimes with different components. In this study we have attempted to model this complexity based on clinical trial data and plausible assumptions.

We compare the projected lifetime costs and effects in the Netherlands of a cohort receiving TPV/r as compared to CPI/r, both administered with an optimized background regimen including at least 2 non-PI antiretrovirals (NRTIs, NNRTIs or enfuvirtide).

## Methods

### Study design

**Figure 1:**
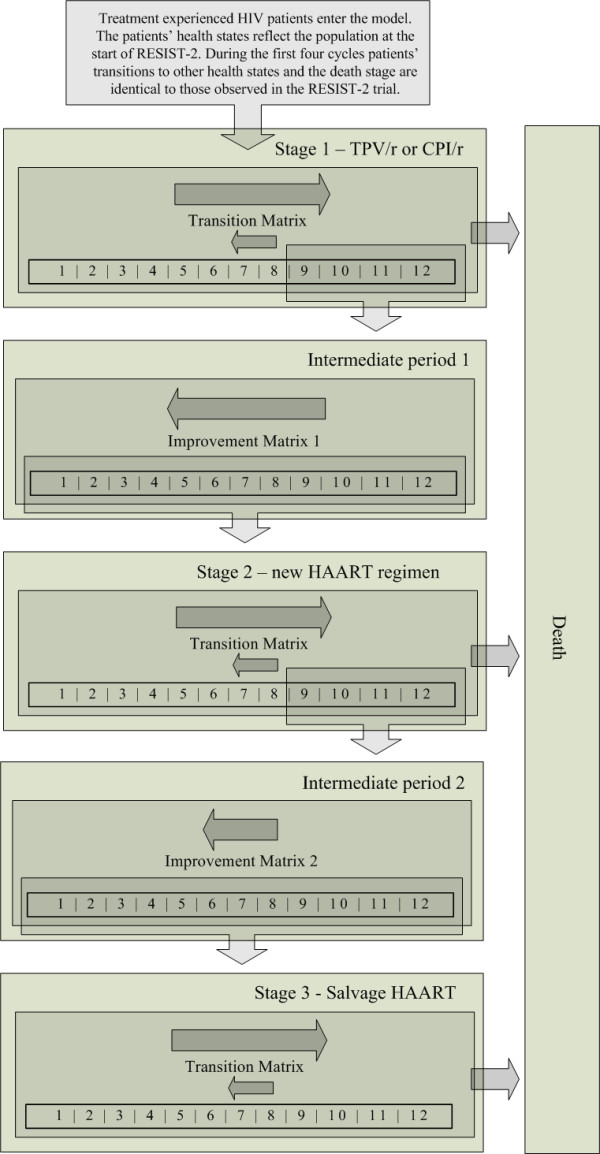
**Diagram of the Markov model**. The model consists of three stages representing three different consecutive therapy regimens. All patients start in stage 1 and are assigned an initial health state based on the population of RESIST-2 at the start of the trial. During the first four cycles of the model patients' transitions to other health states and the death stage are identical to those observed in the RESIST-2 trial. After this trial-period, the patients' health state transitions are controlled by transition matrices based on observational data. The main trend over time is towards a less favorable health state indicated by the larger arrow pointing to the right. When patients reach health state 9–12 (treatment failure), they are moved to stage 2 through intermediate period 1. Patients remain in this intermediate period for 1 cycle where they transit to a more favorable health state controlled by improvement matrix 1, represented by the arrow pointing to the left. Patients move from stage 2 to 3 following the same pattern. TPV/r: tipranavir with ritonavir. CPI/r: comparator protease inhibitor with ritonavir. HAART: highly active antiretroviral therapy.

To assess the life-time costs and effects of a TPV/r based regimen compared to CPI/r, we used two theoretical groups of 1000 HIV-1 infected patients in the Netherlands. One group was assumed to receive a regimen containing TPV/r and the other a CPI/r-containing regimen. All patients had previously been exposed to antiretroviral regimens. A 3-stage Markov model was formulated to simulate the costs and effects during the lifetime of this treatment experienced group of patients. A similar model was previously applied to evaluate the cost-effectiveness of lopinavir/ritonavir versus nelfinavir [16]. A diagram of the model is shown in figure 1. The three stages represent three different consecutive HAART regimens. Patients transit to a next stage if their HAART regimen fails. Treatment failure is determined on the patients' health state. The model uses 12 health states based on viral load and CD4+ count to simulate disease progression (table 1). We use a Markov cycle of three months, corresponding to the average time between patients' visits and the associated analysis of markers for disease progression (CD4+ and viral load parameters). The model runs until 90% of the patients have died ("have entered the absorbing death stage in the Markov model"). The analysis was conducted from a Dutch healthcare perspective, implying that only direct medical costs were taken into account and indirect costs due to production losses were excluded. The analysis meets the most recent Dutch guidelines for pharmacoeconomic research, implying that both costs and effects were discounted at 4% and 1.5%, respectively [17]. All costs were measured in Euros using 2006 price levels.

**Table 1:** Health states with associated risks of AIDS events and quality of life weights

**Heath State**	**CD4+ count^1^**	**Viral load^2^**	**AIDS events^3 ^[18]**	**Quality of Life weight [19]**
1	>500	Undetectable	1.71	0.954
2	>500	Detectable	2.18	0.938
3	351–500	Undetectable	1.71	0.934
4	351–500	Detectable	2.18	0.931
5	201–350	Undetectable	2.84	0.929
6	201–350	<10,000	3.31	0.931
7	201–350	=>10,000	4.25	0.933
8	50–200	Undetectable	5.11	0.863
9	50–200	<10,000	5.58	0.865
10	50–200	10,000–100,000	9.79	0.826
11	50–200	>100,000	14.47	0.876
12	<50	Any viral load	17.87	0.781

The 12 health states defined by CD4+ counts and viral load. Also shown are the associated risks of AIDS events and quality of life (QOL) weights for each health state.

^1 ^Number of CD4+ cells per micro liter

^2 ^RNA copies/mL (as measured by the Roche Amplicor HIV-1 Monitor)

^3 ^Number of events per 100 patients over a 3-month period

### Details on the model

#### Model transitions

Patients transit to a next model stage if their HAART regimen fails. This is determined by the patient's health state. Patients may transit both to more and less favorable health states. However, the main trend is progression to a less favorable health state. During all three model stages, if a patient reaches one of the treatment failure health states (9, 10, 11 and 12), the patient moves to the next Markov stage. Treatment failure is exactly defined as reaching a detectable viral load and a CD4+ count of <= 200. A patient moves to the next Markov stage through an intermediate period of improvement where the patient remains one Markov cycle. In this intermediate period, patients progress to a more favorable health state.

As mentioned above, the analysis was conducted on a theoretical cohort provided with two alternative treatment regimens. The cohort was comparable to the study participants in the RESIST-2 trial with respect to viral load and CD4+ count (see appendix for a brief description of the trial [2]). In particular, all patients start in model stage 1 and are assigned a starting health state reflecting the viral load and CD4+ counts of the population of RESIST-2 at the start of the trial. During the initial 4 cycles the patients follow transitions to other health states and the death stage as observed during the first 48-week of the RESIST-2 trial, based on the patient's last viral load measurement and the average of the CD4+ measurements of each 3-month period. Patients in both arms of the RESIST-2 trial that dropped out were considered as patients failing on treatment and progressed to the second stage of the model (causes for dropping out were among others 'no confirmed 1 log drop in viral load from baseline', 'virological failure', and 'treatment discontinuation due to adverse events'). Since data from the RESIST-2 trial was available for this analysis up to 48-weeks, patient transitions after this period were modeled using transition matrices that were derived from a large observational cohort of 1546 HAART-experienced patients in the United States [18] representing the quarterly progression rates observed for patients that have spent at least 6 months on HAART. These matrices contain the probability of progression or regression of the patient to the other health states and to the death stage. The improvement in intermediate periods 1 and 2 is governed by improvement matrices 1 and 2, which were also derived from the aforementioned observational cohort [18]. These two improvement matrices of the intermediate periods represent the improvement in CD4+ cell count and suppression in viral load that occur when a failing patient is switched to a new HAART regimen; i.e. a proportion of patients is boosted into a more favorable health state. Improvement matrix 1 of intermediate period 1 reflects the immediate response to a new HAART regimen: 70% of the patients were assumed to have an increase of 50 CD4 cells/μl and 20% were assumed to achieve an undetectable viral load. The remaining 10% were unchanged. The improvement matrix 2 of intermediate period 2 reflects the immediate response to a salvage HAART regimen: 30% of the patients were assumed to have an average increase of 30 CD4 cells/μl and 10% of patients were assumed to achieve an undetectable viral load. The remaining 60% were unchanged.

#### Therapy regimens

In stage 1 of the model one group receives TPV/r and the other CPI/r. The different CPIs used – taken from the RESIST-2-trial – are shown in table 2. TPV/r or CPI/r were part of at least a triple therapy regimen consisting of different antiretrovirals, hereafter called the background regimen. The components of the background regimen used – also taken from the RESIST-2-trial – are listed in table 3. In stage 2 patients receive a new HAART regimen. This regimen consists of 2 of the PIs listed in table 2 combined with lamvudine and stavudine. In stage 3 all patients receive a salvage regimen. Various salvage regimens can be considered. For costing, we considered a regimen that is similar to the previous HAART regimen but with a doubled dose of stavudine.

**Table 2:** Daily cost per regimen

**Regimen**	**Daily cost (€)**
Stage 1 – TPV/r + background antiretrovirals	71.70
Stage 1 – CPI/r + background antiretrovirals	55.74
Stage 2 – New HAART regimen	44.04

Stage 3 – Salvage HAART regimen	51.22

Daily cost (Euro price level 2006) per regimen in the Netherlands

TPV/r: tipranavir with ritonavir

CPI/r: comparator protease inhibitor with ritonavir

HAART: highly active antiretroviral therapy

**Table 3:** Daily cost of regimen components

**Protease inhibitor**	**Daily costs (€)**
tipranavir/ritonavir	28.00
**Comparator protease inhibitor**	
lopinavir/ritonavir	14.54
amprenavir	15.21
Indinavir	8.93
Saquinavir	8.58
**Background antiretroviral**	
Abacavir	9.54
Didanosine	6.96
Efavirenz	8.68
Emtricitabine	6.49
Enfuvirtide	25.27
Lamivudine	6.20
Nevirapine	7.79
Stavudine	5.38
Tenofovir	11.70
Zidovudine	8.04

Daily cost (Euro price level 2006) in the Netherlands of tipranavir, comparator protease inhibitors and antiretroviral background regimen components in the RESIST-2 trial (source: Z-index Nov. 2006) [20].

#### AIDS events and quality of life

Each health state corresponds to an associated risk of an AIDS event (table 1). For each health state the risk of a new AIDS event within a 3-month period was derived from the observational cohort [18]. It was assumed that only one type of event can occur to the same patient in each cycle. Additionally, each health state has an associated quality of life (QOL) weight. These were derived from the analysis of data from about 21,000 clinical trial HIV patients assessed with the EuroQol instrument [19]. Each EuroQol-measurement within 30 days of a viral load measurement and CD4+ cell count was classified into one of the 12 health states. The average quality weight per health state was subsequently calculated. Next, the model was run for both the TPV/r and CPI/r groups and life years were counted. The difference between both arms gave the life years gained (LYG). Weighting the life years with the respective quality-of-life weights gave the gains in quality adjusted life years (QALYs).

### Costs

In the first model stage, the costs of the PI regimens TPV/r and CPI/r differ while the cost of the background antiretrovirals in stage 1 are the same in both groups. For stages 2 and 3, the regimen and the associated costs do not differ between groups. The regimen costs of therapy in each model stage are shown in table 2. These regimen costs are the weighted average of the daily costs of individual regimen components, shown in table 3, based on unit costs [20] and utilization in RESIST-2 (unpublished data). The costs of HAART regimens were varied in sensitivity analysis.

During all model stages and in both treatment groups the same assumptions were used on costs and resource use of HIV care. Patients were assumed to routinely visit the clinic every three months corresponding to the average time between patients' visits in the Netherlands. The costs of these visits were assumed at €351, including a CD4+ cell count (€38) and viral load measurement (€209). Additional costs of switching to a different antiretroviral regimen were also assumed at €351. The extra costs of changes in HAART regimen due to toxicities and the treatment of toxicities were not included in the model.

The resource utilization and associated costs for AIDS events (22 most common opportunistic infections and other HIV events) were based on data from the University Medical Centre Groningen (UMCG). The weighted average cost of the different types of AIDS events was estimated at €8,264, using a bottom-up approach using resource use data from the UMCG of the different types of AIDS events and their relative occurrence, shown in table 4. This weighted average was adjusted to take into account that for a number of AIDS event types no reliable cost data was available from the UMCG databases due to the relatively small number of HIV patients (N = 400). In particular, economic data was gathered for resource use concerning AIDS events and for corresponding unit costs. Initially, for all HIV-positive patients in the UMCG that experienced one or more of such events in the years 2001 to 2004 direct medical resource use data was extracted from the UMCG databases, 2 weeks before up to 5 weeks after the registered date of the HIV-related event. Next, this resource use was manually verified and corrected by a research nurse using the electronic and paper patient medical dossiers (permit of the Hospital Ethical Committee was acquired). Incomplete patient files were excluded, resulting in 36 successfully analyzed AIDS events. Resource use was valued using the Dutch guidelines on pharmacoeconomic research [17] where possible. In case costing data was missing, we used reimbursement prices using Dutch tariffs [21].

**Table 4:** Occurrence and estimated cost of AIDS events

**AIDS event**	**Relative occurrence (%)**	**Number observed in UMCG (N)**	**Estimated Cost (€)**
Candidiasis, oral or systemic	9.0	7	5,642
Herpes simplex	0.3	5	6,615
Kaposi sarcoma, cutaneous	3.8	1	1,972
Lymphoma	3.0	3	21,640
Mycobacterium avium complex	5.8	1	14,733
Pneumocystic pneumonia	13.0	7	8,214
Pneumonia	20.1	4	9,568
Tuberculosis	4.9	4	12,432
Toxoplasmic encephalitis	1.9	2	16,822
Wasting syndrome	22.8	1	4,072
Cryptococcal meningitis	1.2	1	11,354
Other	14.2	0	n.a.

Total	100	36	8,264

Occurrence of AIDS events and estimated costs (Euro price level 2006) as observed in the UMCG. For 14.2% of all AIDS events no event data was available in the UMCG due to the relatively small number of patients. These types are Cervical cancer, Cytomegalovirus Retinitis, Cytomegalovirus Other, Coccidiosis, HIV-Dementia, Histoplasmosis, Kaposi sarcoma, visceral, Salmonella sepsis, Cryptosporidium, Progressive multifocal leucoencephalopathy

UMCG: university medical center Groningen

### Sensitivity analysis

We assessed the uncertainty in the model parameters through univariate sensitivity analysis. All cost parameters were varied in the univariate sensitivity analysis over a range of 75% to 125% of the base-case parameter value and the impact on the iCER per QALY was presented in a tornado diagram. The impact of the AIDS event rates were investigated by multiplying these rates by 0.75 and 1.25. Additionally, the discount rates were evaluated at values of 0% and 5%. To assess the impact of a slower or faster disease progression (that is reflected in the transition probabilities of the transition matrices), we varied the probability for each health state of a patient staying in that particular health state by 5%. Simultaneously, the probability of transiting to the other health states is proportionately increased or decreased. Utility weights were not included in the sensitivity analysis.

## Results

### Base-case results

The lifetime accumulated cost to the healthcare system per patient receiving the TPV/r regimen is estimated to be €167,200 compared to €145,400 for a patient receiving the CPI/r regimen. This results in an incremental cost per patient of €21,800 (including lifetime savings of €167 per patient due to the reduction in AIDS events). The accumulated effect is 7.43 life years or 6.31 QALYs per patient receiving TPV/r, compared to 6.91 life years or 5.80 QALYs per patient receiving CPI/r. This translates into an incremental effect per patient receiving TPV/r compared to CPI/r of 0.52 LYG or 0.51 QALYs gained per person. The equivalent incremental cost effectiveness ratios (iCERs) are €41,600 per LYG and €42,500 per QALY.

### Sensitivity analysis

**Figure 2:**
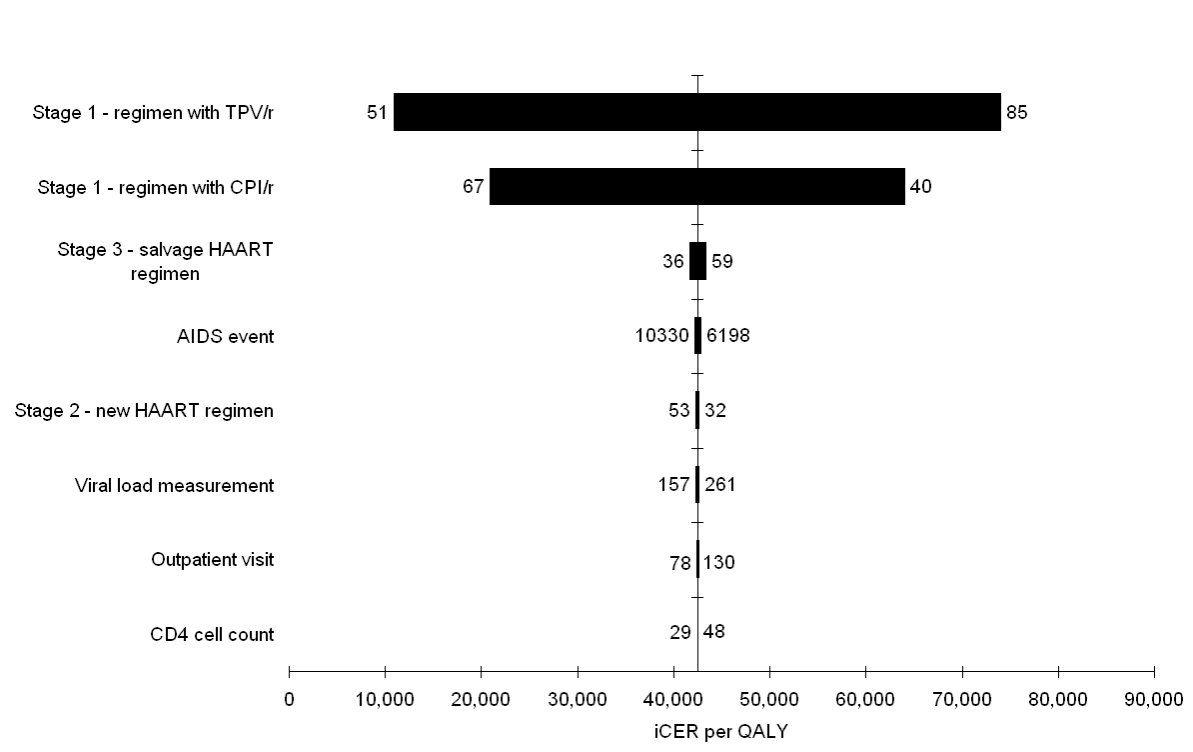
**Tornado diagram of the sensitivity analysis**. Tornado diagram of the univariate sensitivity analysis showing the impact of individual cost parameters on the iCER per QALY. Parameter values of 75% and 125% of the base-case value were evaluated and these values are shown on both sides of the bars. All parameters refer to costs and are expressed in Euro price level 2006. TPV/r tipranavir with ritonavir. CPI/r comparator protease inhibitor with ritonavir. HAART highly active antiretroviral therapy. iCER incremental cost effectiveness ratio. QALY quality adjusted life year.

The results of the univariate sensitivity analysis on cost parameters are presented in figure 2. This tornado diagram ranks the cost parameters based on the magnitude of their impact on the iCER per QALY. The diagram shows clearly that the regimen costs of both arms have the highest impact on the model outcome. For the remaining cost parameters, the model proved to be robust to changes. Multiplying all AIDS event rates simultaneously by 0.75 and 1.25 had a relatively low impact on the iCER of €42,800 and €42,200 per QALY, respectively. Additionally, the discount rates were evaluated at 0% and 5%. This resulted in €51,600 and €40,700 per QALY for the discount rate for costs, and €37,900 and €54,300 per QALY for the discount rate for effects, respectively. The impact of a 5% slower and faster disease progression on the iCER was modest with €41,300 and €43,700 per QALY, respectively.

## Discussion

Our model generally shows that TPV/r versus CPI/r treatment in heavily treatment experienced HIV infected patients provides benefits regarding reductions in AIDS events and corresponding QALY gains and life years saved. This was achieved at a cost-effectiveness ratio of crudely €40,000 per LYG or QALY.

Generally in the Netherlands drug interventions with an iCER below €20,000 per LYG are certainly considered cost-effective and are accepted for reimbursement [22]. In particular in the field of last-resort treatments however – including inpatient oncologic treatments and HIV treatments – drugs with an iCER over €20,000 per LYG are accepted. Various considerations underlie this: (i) in the international context the Dutch cost-effectiveness threshold at €20,000 is relatively low, compared to for example to the generally accepted $50,000 per QALY for the United States or £30,000 for the United Kingdom; and (ii) recent discussion at Ministerial level have suggested to raise the Dutch threshold up to €80,000. Our base-case estimate for TPV/r may thus well be acceptable in the Dutch context. Indeed, tipranavir was recommended for reimbursement in the Netherlands by the Foundation for Health Care Insurance in February 2006. In a budget impact analysis, this institution estimated the additional costs of treatment with TPV/r in the Netherlands to be €905,000 €1,470,000 and €2,070,000 for the first, second and third year respectively, assuming a number of HIV patients on HAART in the Netherlands of 7747 and a substitution of 30% within the patient population eligible for TPV/r. The number of PI-experienced patients on salvage treatment and eligible for TPV/r was estimated to be no higher than 235 in the 3^rd ^year [23].

In the context of the international health economic literature for treatment of HIV disease, cost-effectiveness results vary significantly. These studies generally do however not directly compare two antiretroviral components in the context of HAART. In the UK, Miners et al. reported an iCER of UK £17,698 per QALY in 2000 for adding an unspecified PI to lamivudine and zidovudine [24]. Anis et al. compared triple therapy to two dual regimens, reporting iCERs of Can $46,971 and Can $58,806 per LYG in 1997 for the stavudine- and zidovudine-based regimens, respectively [25]. In the context of HAART, Simpson et al. have evaluated lopinavir/ritonavir versus nelfinavir and report an iCER per QALY of US $6,653 in 2002 using the same model as was used for this study based on a different clinical trial [16]. Caro et al. report efavirenz to be cost saving when comparing efavirenz versus indinavir combined with two NRTIs in 1998 [26].

We have used a Markov model to approximate the complexity of sequential antiretroviral regimens that are typical for treatment of HAART experienced patients. In the absence of transition probabilities for the Netherlands, our study is limited by the fact that the health state transition probabilities were based on a clinical trial with primarily non-Dutch participants and a large observational cohort in the United States. Also, the AIDS event rate for each health state was calculated from this cohort. However, transition probabilities and AIDS event rates found comparable to those derived from a cohort of 3000 US patients and 900 Dutch patients, published by Ghani and colleagues [27]. Additionally, AIDS event cost data was collected from a Dutch population of HIV infected patients treated in the UMCG, whereas the majority of HIV infected patients in the Netherlands are treated in Amsterdam. We do however not expect major differences between the Amsterdam and Groningen patient populations.

We did not specifically include the costs of adverse events in this model because inclusion of such costs in this same analysis for other countries (unpublished data) had shown that this inclusion had a very minor effect on the iCER. We note that our estimate for the cost of an AIDS event of €8,264 is substantially lower than the $30,291 (€30,466 price level 2006) reported by Simpson et al. for the United States in 2002 [16]. This difference can be partially attributed to the 5-week cut-off after the date of the AIDS-event used for the resource use data collection. Our approach reflects a conservative approach, neglecting chronic treatment or potential long-term complications of which the direct relation with the event may be very uncertain. Also, this model parameter has a very limited effect on the iCER. Using the value reported by Simpson et al. [16] our model projects an iCER of 39,000 €/QALY, just slightly below our base-case estimate.

## Conclusion

In conclusion, we estimated the iCER per QALY for TPV/r compared to CPI/r to be €42,500 in treatment experienced HIV-1 infected patients in the Netherlands. This ratio may well be in range of what is acceptable and warrants reimbursement for new drug treatments in the Netherlands, in particular in therapeutic areas as end-stage oncology and HIV and other last-resort healthcare interventions.

## Competing interests

This study was facilitated by an unrestricted grant from Boehringer-Ingelheim.

## Authors' contributions

GAAH is the main investigator of this study and corresponding author. JMB contributed to the manuscript and provided the sensitivity analysis. CAVS and SS performed the acquisition of data from the UMCG. HWF critically reviewed several versions of the manuscript. BSK and AT have provided information on the clinical aspects of HIV infection and have contributed to the manuscript. KNS is the designer of the mathematical model and provided all transition data. MJP has supervised this study.

## Appendix – Summary of the RESIST-2 trial and its 48-week results

The population of the randomized phase III efficacy and safety trial RESIST-2 consisted of HIV-1 positive patients in Europe and Latin America that have experienced previous treatment from all classes of antiretrovirals: Nucleoside Reverse Transcriptase Inhibitors, Non Nucleoside Reverse Transcriptase Inhibitors and at least two protease inhibitor based regimens for a period of minimum three months. The trial was conducted over the period 2003–2004. Patients had a viral load of >= 1000 with any CD4+ cell count, with at least one primary PI mutation. The baseline mean and standard deviation (between brackets) of CD4+ count was 219 (192) for the TPV/r arm and 217 (168) for the CPI/r arm. The overall aim of the study was to investigate whether TPV demonstrates similar or better efficacy and comparable safety as the drugs chosen in the active control group. The endpoint was the proportion of patients with a treatment response in terms of at least a 1 log_10 _viral load reduction.

All patients received an optimized standard of care regimen selected by their physician. Patient were randomized to include in this regimen either TPV/r or a comparator PI/r.

A total of 879 patients were randomized, with 863 being evaluable. Kaplan-Meier estimates of time to treatment failure indicated a significant difference in favor of TPV (p < 0.0001). Treatment response after 48 weeks was higher in the TPV group at 34% versus 15% in the active controls. Many active controls (57%) changed the drug or discontinued due to virologic failure (versus 18% in the TPV group). In the different strata defined by comparator drug (lopinavir, saquinavir and amprenavir) similar results were seen. Also, CD4+ counts increased more for TPV than for controls (26 cells/mm^3 ^versus 1 cell/mm^3^), with similar counts at baseline (219 and 217). Co-administration of enfuvirtide increased the proportion of patients with a treatment response in both arms of the trial. The researchers summarize that: "the results of this 48-week interim summary show that the TPV regimen (in combination with other antiretroviral agents) was significantly more effective than the comparator regimen (in combination with other antiretroviral agents) in postponing treatment failure or achieving a treatment response in patients who had previously received multiple antiretroviral therapy". Benefits provided by the TPV regimen through 48 weeks were enhanced by co-administration of other active antiretroviral agents, including enfuvirtide. Adverse events (AEs) occurred more in the TPV arm of the trial (88% vs. 78% of patients for all AEs). This resulted in discontinuation of study medication for 11% of TPV users and 6% for comparator drugs. There were no differences in overall serious AEs (both at 17%), however 3% of TPV users had serious AEs considered related to study medication, against 0.5% for comparator drugs.
